# Meta-Analysis on the Safety and Cardiorenal Efficacy of SGLT2 Inhibitors in Patients Without T2DM

**DOI:** 10.3389/fcvm.2021.690529

**Published:** 2021-06-30

**Authors:** Lu-Feng Li, Liang-Liang Ding, Ze-Lin Zhan, Mei Qiu

**Affiliations:** ^1^Department of General Medicine, Shenzhen Longhua District Central Hospital, Shenzhen, China; ^2^Department of Endocrinology, First Affiliated Hospital of Yangtze University, Jingzhou, China; ^3^Class 3, Clinical Medicine, Grade 2019, The Second Clinical Medical College, Southern Medical University, Guangzhou, China

**Keywords:** SGLT2 inhibitors (gliflozins), heart failure, chronic kidney disease, type 2 diabetes mellitus, death

## Abstract

The cardiorenal benefits of sodium-glucose cotransporter 2 (SGLT2) inhibitors in patients with type 2 diabetes mellitus (T2DM) are established, whereas those in patients without T2DM are not established. We sought to assess the cardiorenal efficacy and safety of SGLT2 inhibitors in non-T2DM patients by performing a meta-analysis based on the subgroup data of non-T2DM patients from relevant secondary analysis articles in which subgroup analyses were done according to the status of diabetes. Compared to placebo, SGLT2 inhibitors significantly reduced heart failure hospitalization [risk ratio (RR) 0.70, 95% confidence interval (CI) 0.59–0.83] and kidney-specific composite outcome (RR 0.55, 95% CI 0.40–0.75) and increased Kansas City Cardiomyopathy Questionnaire total score by 1.15 (95% CI 1.05–1.25) in patients without T2DM with heart failure (HF) or chronic kidney disease (CKD), whereas gliflozins did not significantly affect cardiovascular death, all-cause mortality, volume depletion, fracture, and amputation in this vulnerable population. There was no event of major hypoglycemia or diabetic ketoacidosis observed in the non-T2DM subgroup in included trials. These findings will further prompt gliflozins to be used for the prevention of HF and renal failure events and for the improvement of life quality in patients without T2DM with HF or CKD.

## Introduction

According to the evidence from large trials assessing the effects of sodium-glucose cotransporter 2 (SGLT2) inhibitors on cardiovascular or renal outcomes, the cardiorenal benefits of SGLT2 inhibitors have already been extended from patients with type 2 diabetes mellitus (T2DM) (1–5) to patients with heart failure (HF) (6, 7) and those with chronic kidney disease (CKD) (8). Accordingly, both the updated consensus report (9) from the American Diabetes Association (ADA) and European Association for the Study of Diabetes (EASD) and the guidelines (10) from the European Society of Cardiology (ESC) propose that SGLT2 inhibitors should be used for prevention of cardiorenal adverse endpoints in T2DM patients with HF or CKD or in those at high cardiorenal risk (11). Conversely, the cardiorenal benefits of SGLT2 inhibitors in patients without T2DM are not established because there are no large randomized trials which have focused on assessing cardiorenal endpoints with SGLT2 inhibitors in non-T2DM patients.

Three large trials (6–8), aiming to evaluate the effects of dapagliflozin or empagliflozin on cardiorenal outcomes in patients with CKD or HF, involved a certain proportion of non-T2DM patients. Moreover, the outcome data of gliflozins in non-T2DM patients have recently been reported in three articles (12–14) in which subgroup analyses were conducted according to the status of diabetes. However, these individual subgroup analysis articles (12–14) are not powered to evaluate the effects of gliflozins in non-T2DM patients. Hence, we intended to perform a meta-analysis incorporating the subgroup data from non-T2DM patients in order to assess the safety and cardiorenal efficacy of SGLT2 inhibitors in this vulnerable population.

## Methods and Findings

Because there are no relevant randomized trials which have specially assessed the safety and cardiorenal endpoints with SGLT2 inhibitors in non-T2DM patients, we conducted this meta-analysis using the subgroup data of non-T2DM patients which were derived from three recent articles (12–14) reporting the subgroup analyses according to the status of T2DM. The six efficacy endpoints we assessed in this meta-analysis were as follows: a composite of hospitalization for heart failure (HHF) or cardiovascular death (CVD), CVD, HHF, all-cause mortality (ACM), kidney-specific composite outcome (KSCO), and change in Kansas City Cardiomyopathy Questionnaire (KCCQ) total score. The eight safety endpoints of interest were the following: any serious adverse event, discontinuation of study drug due to adverse event, volume depletion, kidney adverse event, fracture, amputation, major hypoglycemia, and diabetic ketoacidosis.

Two authors separately extracted the outcome data of the non-T2DM subgroup from the included trials. The included trials were assessed for risk of bias according to the Cochrane risk of bias assessment tool (15). Any inconsistencies between them were solved by the arbitrament of a third author. To perform meta-analysis, we extracted mean difference (MD) and 95% confidence interval (CI) for the outcome of change in KCCQ total score, while we extracted dichotomous data for all the other outcomes and utilized risk ratio (RR) and 95% CI as drug effect. We performed both random-effects and fixed-effects meta-analysis. When the *I*^2^ statistic was >50% we selected the random-effects results, and when the *I*^2^ statistic was ≤ 50% we selected the fixed-effects results.

The outcome data in the non-T2DM subgroup were extracted from the three trials: Dapagliflozin and Prevention of Adverse Outcomes in Chronic Kidney Disease (DAPA-CKD (12) trial enrolling CKD patients with/without T2DM, and EMPagliflozin outcomE tRial in Patients With chrOnic heaRt Failure With Reduced Ejection Fraction (EMPEROR-Reduced) (13) and DAPA-HF (14) trials enrolling HF patients with/without T2DM. The three trials were all high-quality studies with low risk of bias and involved a total of 5,871 non-T2DM patients consisting of 2,928 taking gliflozins vs. 2,943 taking a placebo.

**Figure 1:**
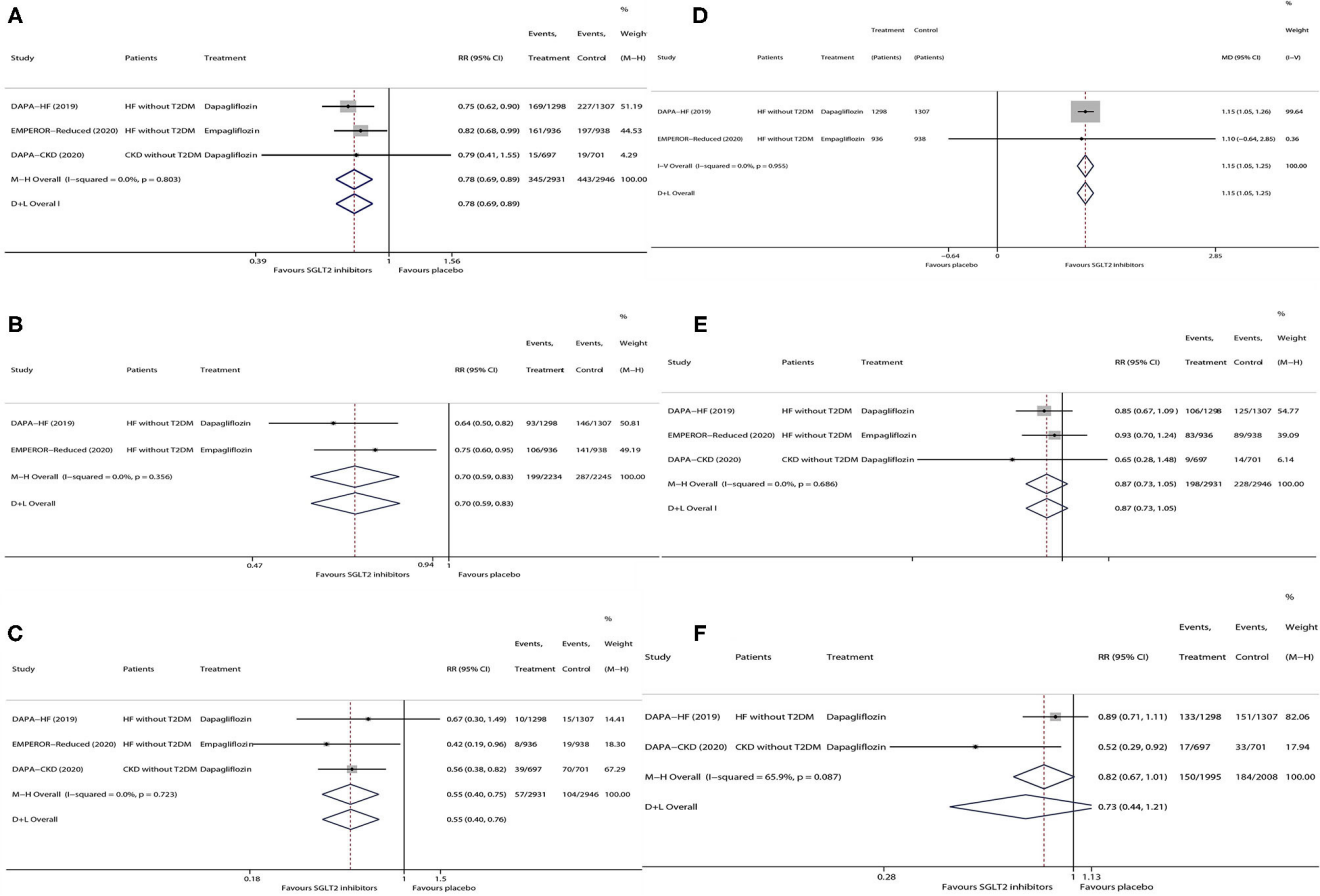
Meta-analysis of the effects of SGLT2 inhibitors on HHF or CVD **(A)**, HHF **(B)**, KSCO **(C)**, change in KCCQ total score **(D)**, CVD **(E)**, and ACM **(F)** in patients without T2DM. SGLT2, sodium-glucose cotransporter 2; HHF, hospitalization for heart failure; CVD, cardiovascular death; KSCO, kidney-specific composite outcome; KCCQ, Kansas City Cardiomyopathy Questionnaire; ACM, all-cause mortality; HF, heart failure; T2DM, type 2 diabetes mellitus; CKD, chronic kidney disease; MD, mean difference; RR, risk ratio; CI, confidence interval.

As is shown in Figure 1, in patients without T2DM with HF/CKD, SGLT2 inhibitors vs. placebo significantly lowered the risks of HHF or CVD (RR 0.78, 95% CI 0.69–0.89; Figure 1A), HHF (RR 0.70, 95% CI 0.59–0.83; Figure 1B), and KSCO (RR 0.55, 95% CI 0.40–0.75; Figure 1C) and increased KCCQ total score by 1.15 (95% CI 1.05–1.25; Figure 1D), whereas SGLT2 inhibitors did not have significant effects on the risks of CVD (RR 0.87, 95% CI 0.73–1.05; Figure 1E) and ACM (RR 0.73, 95% CI 0.44–1.21; Figure 1F).

**Figure 2:**
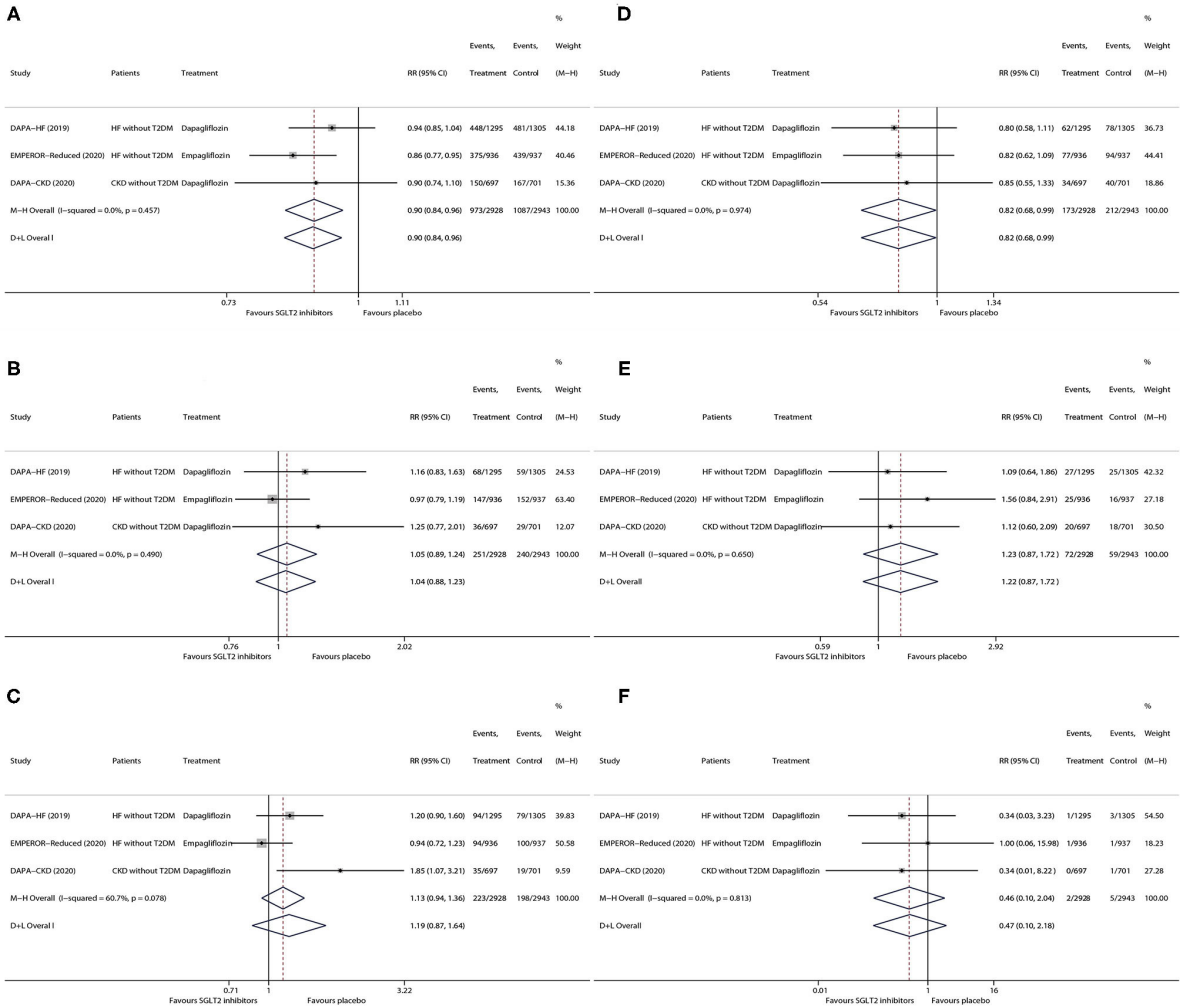
Meta-analysis of the effects of SGLT2 inhibitors on any serious adverse event **(A)**, discontinuation of study drug due to adverse event **(B)**, volume depletion **(C)**, kidney adverse event **(D)**, fracture **(E)**, and amputation **(F)** in patients without T2DM. SGLT2, sodium-glucose cotransporter 2. HF, heart failure; T2DM, type 2 diabetes mellitus; CKD, chronic kidney disease; RR, risk ratio; CI, confidence interval.

As is shown in Figure 2, in patients without T2DM with HF/CKD, SGLT2 inhibitors vs. placebo significantly lowered the risks of any serious adverse event (RR 0.90, 95% CI 0.84–0.96; Figure 2A) and kidney adverse event (RR 0.82, 95% CI 0.68–0.99; Figure 2D), whereas SGLT2 inhibitors did not have significant effects on the risks of discontinuation of the study drug due to adverse event (RR 1.05, 95% CI 0.89–1.24; Figure 2B), volume depletion (RR 1.19, 95% CI 0.87–1.64; Figure 2C), fracture (RR 1.23, 95% CI 0.87–1.72; Figure 2E), and amputation (RR 0.46, 95% CI 0.10–2.04; Figure 2F). There was no one event of major hypoglycemia or diabetic ketoacidosis observed in the non-T2DM subgroup in all the included trials.

## Discussion

This is the first meta-analysis that fully assessed the safety and cardiorenal efficacy of SGLT2 inhibitors in patients without T2DM. Accordingly, this meta-analysis revealed that SGLT2 inhibitors vs. placebo significantly reduced HHF and KSCO and increased KCCQ total score in patients without T2DM and did not significantly affect CVD and ACM, while SGLT2 inhibitors lowered the risks of serious adverse event and kidney adverse event in this vulnerable population and did not lead to the increased risks of volume depletion, fracture, amputation, major hypoglycemia, and diabetic ketoacidosis.

Several previous meta-analyses (16–20) confirmed the cardiorenal benefits of SGLT2 inhibitors in T2DM patients, while some other meta-analyses (21–23) confirmed those in HF patients. However, these meta-analyses (16–23) failed to specially evaluate the effects of gliflozins in the non-T2DM subgroup. Another meta-analysis (24) focused on assessing the effects of gliflozins in the non-T2DM patients but only assessed one cardiovascular outcome (i.e., a composite of HHF or CVD) and failed to assess the other critical cardiorenal outcomes as well as safety outcomes. In contrast, in this meta-analysis we performed the most comprehensive analysis regarding the safety and cardiorenal effects of gliflozins in non-T2DM patients and therefore confirmed the good safety and cardiorenal benefits of gliflozins in the non-T2DM subgroup. Gliflozins have been recommended in T2DM patients with HF/CKD or at high cardiorenal risk by both the ESC guidelines (10) and the updated ADA–EASD consensus report (9). However, the ESC guidelines (10) have proposed a knowledge gap whether SGLT2 inhibitors lower the risk of HF in non-T2DM patients. Fortunately, the present meta-analysis fills this knowledge gap, and moreover the findings of this meta-analysis suggest that gliflozins should be used for the prevention of HF and renal failure events and for the improvement of life quality in patients without T2DM with HF or CKD. Accordingly, these findings may contribute to updating the current ESC guidelines (10).

Although SGLT2 inhibitors were initially only considered as glucose-lowering agents, the effects of gliflozins have expanded far beyond that, and this drug class has been proven to have pleiotropic metabolic and direct nephroprotective and cardioprotective effects (25). Moreover, SGLT2 inhibitors exert cardioprotective and nephroprotective effects by reducing oxidative stress, inflammation, fibrosis, sympathetic nervous system activation, and intraglomerular hypertension and improving myocardial efficiency and mitochondrial function (25). These above mechanisms of activity of gliflozins appear to have little connection with the underlying disease of T2DM, and therefore the cardiorenal benefits of SGLT2i probably exist in non-T2DM patients too.

This study has two main limitations. First, we assessed the safety and efficacy of gliflozins in overall non-T2DM patients but failed to assess those in more specific subgroups, such as the subgroup of non-T2DM patients with HF and the subgroup of non-T2DM patients with CKD, due to the limited number of included studies. Second, in this meta-analysis SGLT2 inhibitors were not observed to significantly affect CVD and ACM but showed the possibly reduced trends in the two death endpoints. Future studies are needed with sufficient statistical power to define these trends.

In conclusion, compared with placebo, SGLT2 inhibitors significantly reduced HHF and KSCO and increased KCCQ total score in patients without T2DM with HF or CKD, without increasing the risks of volume depletion, fracture, amputation, major hypoglycemia, and diabetic ketoacidosis. These findings will further prompt gliflozins to be used for the prevention of HF and renal failure events and for the improvement of life quality in this vulnerable population.

## Author Contributions

MQ: design. Z-LZ, L-LD, and L-FL: conduct/data collection. L-LD and MQ: analysis. L-FL and MQ: writing manuscript. All authors contributed to the article and approved the submitted version.

## Conflict of Interest

The authors declare that the research was conducted in the absence of any commercial or financial relationships that could be construed as a potential conflict of interest.

## Abbreviations

SGLT2: sodium-glucose cotransporter 2
T2DM: type 2 diabetes mellitus
HF: heart failure
CKD: chronic kidney disease
ADA: American Diabetes Association
EASD: European Association for the Study of Diabetes
ESC: European Society of Cardiology
HHF: hospitalization for heart failure
CVD: cardiovascular death
ACM: all-cause mortality
KSCO: kidney-specific composite outcome
KCCQ: Kansas City Cardiomyopathy Questionnaire
MD: mean difference
CI: confidence interval
RR: risk ratio.
